# The great escape: A tale of the visiting professor’s Artful Dodge

**DOI:** 10.1002/bco2.66

**Published:** 2021-01-26

**Authors:** John W. Davis

**Affiliations:** ^1^ Editor—BJUI Compass

In January 2001, I was a research scholar on a grant from the American Foundation of Urological Disease (now the Urology Care Foundation, under the American Urological Association) in Norfolk, Virginia at Eastern Virginia Medical School (EVMS). The EVMS residency invited Dr Mani Menon from Henry Ford Hospital in Detroit, Michigan to be our visiting professor. Dr Menon was highly focused on building a minimally invasive radical prostatectomy programme, and at this time he was transitioning from the laparoscopic method to the robotic method—a move that would fundamentally change the practice of radical prostatectomy.

As with most visiting professor events, the residents present cases of interest, and while we certainly discussed a lot of prostate cancer, we thought it would be interesting to throw him a curve ball. We presented a case of hereditary renal cancer that had passed in an autosomal dominant fashion and was known to be associated with other clinical manifestations such as skin fibrofolliculomas, pulmonary cysts, and spontaneous pneumothorax. I think many of you will already recognize this as Birt‐Hogg‐Dubé syndrome. However, in 2001, the renal cancer aspects of the syndrome were just being described, even though early reports on the skin disorders go back to 1977. Suffice it to say, Dr Menon was officially “stumped” on any detailed discussions on Birt‐Hogg‐Dubé syndrome (although he did have some impressive ideas in his differential).

But rather than waving the white flag, or just asking for the next case, Dr Menon did something unexpected and highly memorable. As many politicians do at debates or press conferences—if you don't want to answer the question posed, just answer a different question. So Dr Menon turned the case into a useful discussion and explanation on screening test metrics, specific to the challenges of screening for rare events like Birt‐Hogg‐Dubé syndrome. The key take home messages were that:

1. The sensitivity and specificity of a screening marker (or any test) are somewhat fixed by the clinically acceptable thresholds for calling a test normal or abnormal.
2. The positive and negative predictive values are highly related to the prevalence of the condition being screened or tested for.
3. Therefore, if you want to screen for something rare, you really have to have a highly sensitive marker to result in a useful positive predictive value.

**FIGURE 1:**
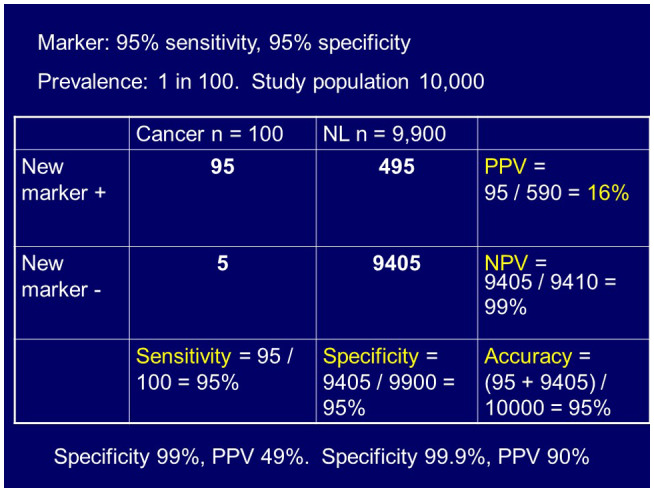
An example of a highly sensitive biomarker, but with low positive predictive value due to low disease prevalence

**FIGURE 2:**
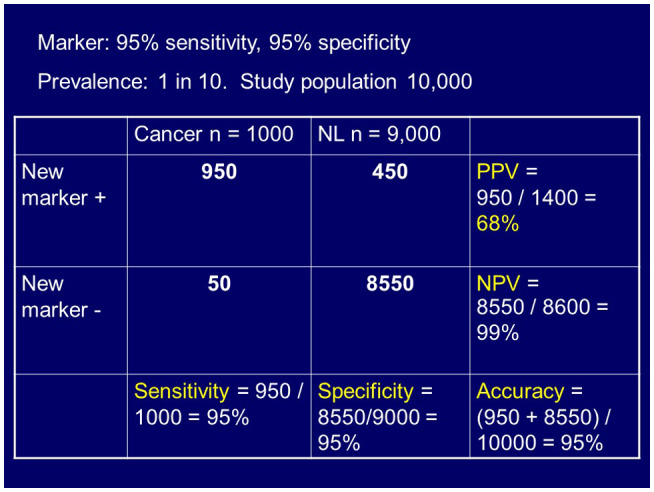
Same testing variables, except that the prevalence is now changed to 1 in 10 with resulting increased positive predictive value

Figures 1 and 2 demonstrate these points, looking at a hypothetical biomarker that has 95% sensitivity and specificity. For Figure 1, you see a prevalence of 1 in 100 and the resulting test metrics show a positive predictive value of 16%. For Figure 2, the variables are similar, but the prevalence is changed to 1 in 10, and a resulting positive predictive value increase to 68%.

**FIGURE 3:**
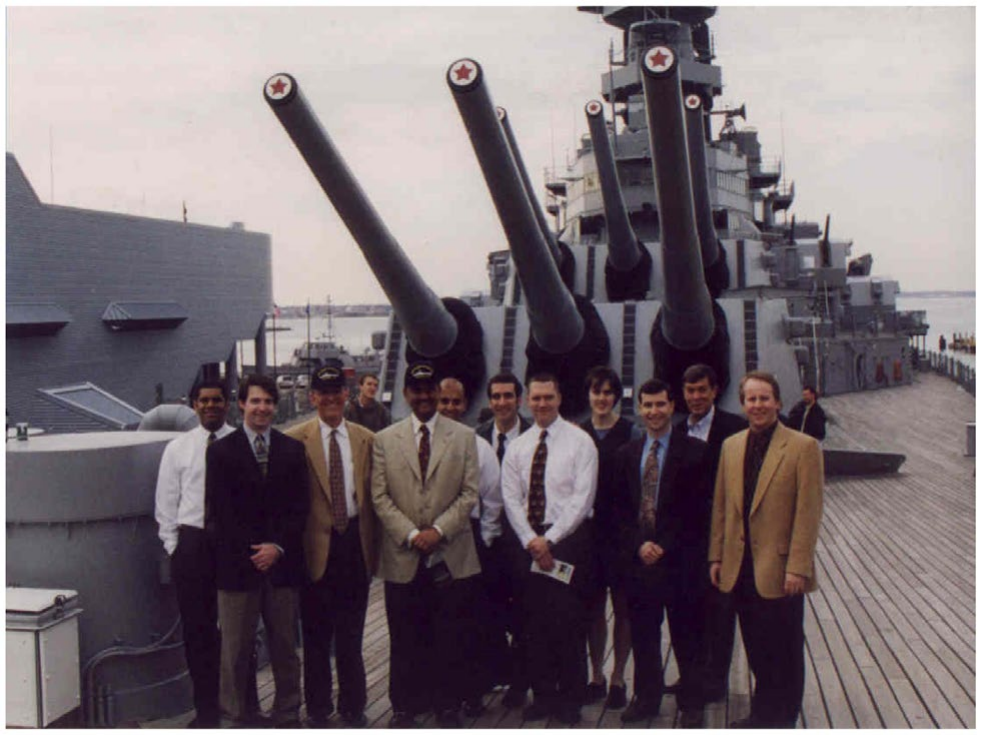
Visiting Professor Mani Menon, January 2001 on the USS Wisconsin at Norfolk, Virginia Harbour

**FIGURE 4:**
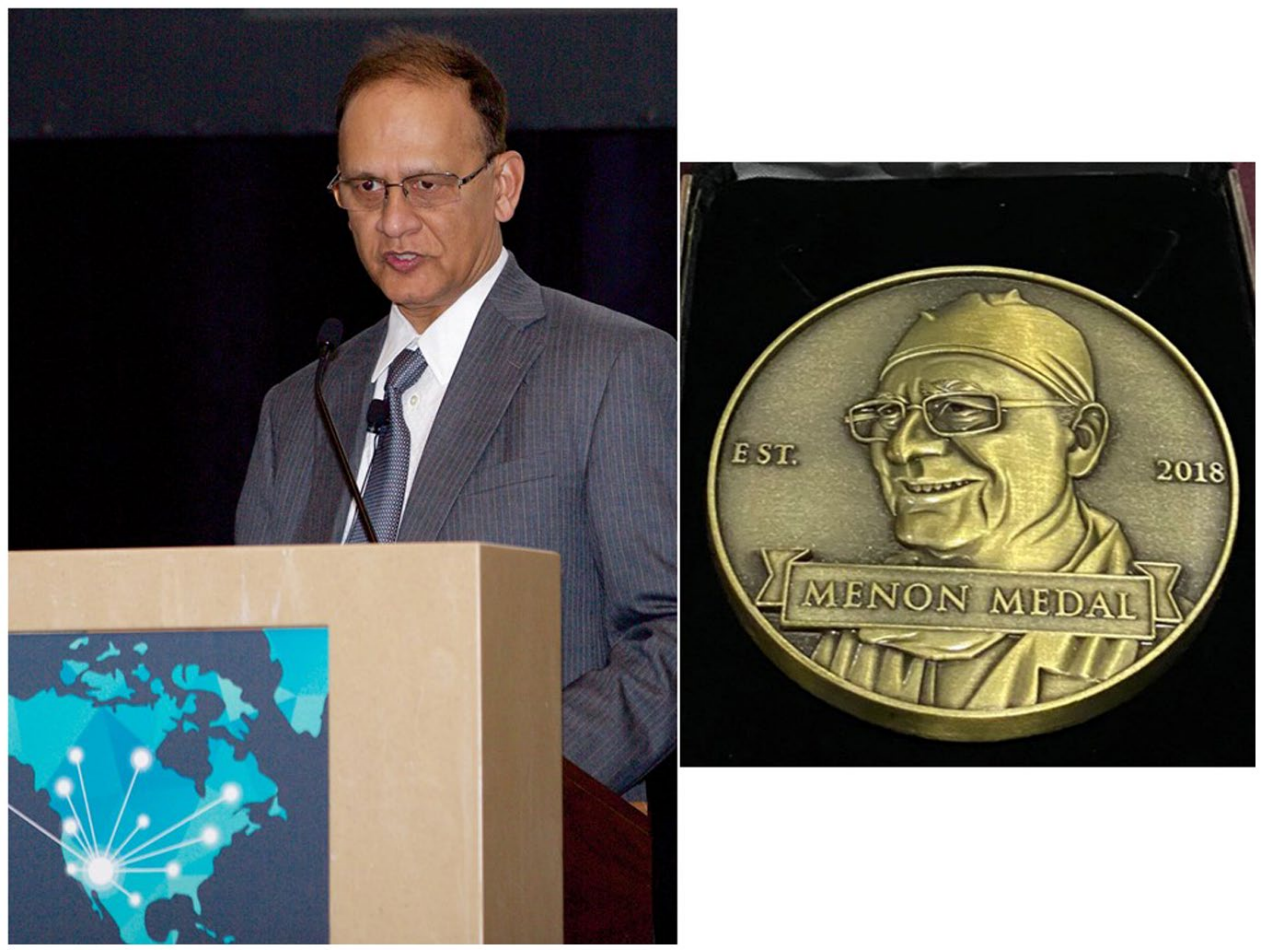
Dr Mani Menon giving his keynote address/award at the 1st North American Robotic Urology Symposium in 2017; an award now known as the Menon Medal

Following this busy day of cases, lectures, and the memorable “artful dodge,” the group moved on with the agenda, and hosted Dr Menon on a tour of the famous battleship Wisconsin that had just been relocated to the Norfolk harbour as a museum; Figure 3 shows are group picture including Dr Menon in the middle with our chair Paul Schellhammer to his right, and your editor on the right side. Fast forwarding to 2017, Dr Menon was recognized by the North American Robotic Urology Symposium in Las Vegas, Nevada for outstanding achievement in robotic surgery (Figure 4), and this award moving forward is now known as the Menon Medal (see www.NARUS.US).

Moving on to the January 2021 issue of *BJUI Compass*, which now features an expanded article count due to increasing volume of quality submissions, and authorship groups from around the globe including the United States, Japan, Australia, and The United Kingdom. One of our noteworthy contributions is from the United Kingdom, Vasdev et al,^1^ on a novel biomarker for urothelial cancer, and hence our theme of screening metrics. The cohort tested is a modest size of 71, but the overall sensitivity was 100% with specificity of 92.6%, and PPV 0f 95.7%. Biomarker development in bladder cancer has been ongoing for many years with a library of options available, but the question has always remained whether or not any of them can truly replace cystoscopy. Again, the prevalence has always been an issue—even with risk targeted populations. Janet Kukreja from our editorial board helped review and optimize this paper, and expands her thoughts on this biomarker in the broader context with an editorial comment.^2^ We look forward to further validation studies.

**FIGURE 5:**
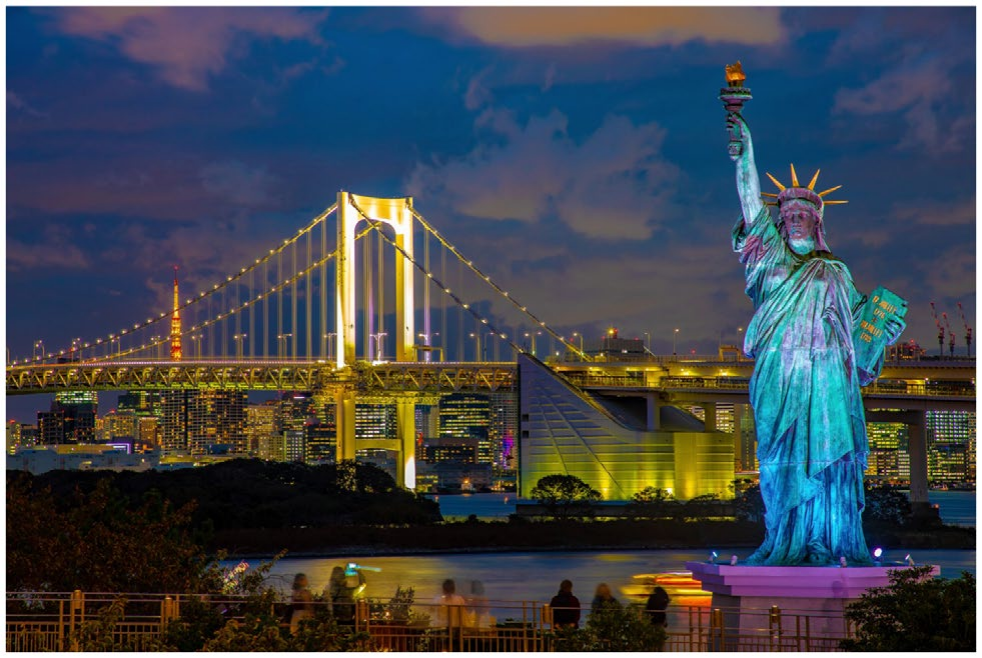
We have three papers this month form Yokohama City, Japan, which is located on Tokyo Bay. Also on the Bay is an interesting statue that will look familiar to Americans, called the Odaiba Statue of Liberty, a tribute to Japan's ties with France

- *To the Journals…* We have one review this issue^3^; a collaboration between United Kingdom and Australian authors on a systematic review/meta‐analysis of ductal adenocarcinoma of the prostate. This is a significant effort to look at all relevant studies: 114 included. The authors found it challenging to do a pure metanalysis, as the data reporting is heterogeneous in histopathologic definitions. They found higher rates of metastatic disease, including unusual sites such as penile and peritoneal. A very recent publication from our centre is now on PubMed and first authored by one of our fellows from Australia, Ranasinghe et al^4^ that goes into additional depth on ductal variant patients and their metastatic patterns from de novo versus progression presentations. Again, showing patterns of multiple sites (bone/viscera), and higher rates of lung metastases.
- *To the Clinic…* We have two articles for the clinic in this issue from Japan. The paper by Miyoshi et al^5^ uses a tool you may or may not be using called an automated bone scan index (aBSI), a tool to objectively quantitate bone metastases burden. They used this index specifically in castrate resistant prostate cancer treated with Radium‐223. They found that the aBSI at baseline and after 12 weeks had prognostic value for overall survival. Continuing with the radium‐223 topic, Miyoshi et al^6^ describe another cohort treated with radium‐223 in combination with abiraterone or enzalutamide, and report the combination is well tolerated in Japanese patients but only a slight trend in benefit. They dive deeper into prognostic observations. With three papers from Yokohama City, Japan in this issue, will give a travel photo shoutout for Figure 5, the Tokyo Bay version of the Statue of Liberty.
- *To the Drawing Board…* We have discussed the Vasdev paper already as part of our editorial theme and this paper will be in our academic section. A second paper, again from the Yokohama City University group in Japan from Iwamoto et al^7^, looked at the problem with sarcopenia as related to metastatic hormone‐naïve prostate cancer. Using CT estimation of lower psoas volume, they could correlate poorer survival and shorter time to castrate resistance in groups with lower psoas muscle index density. I think it is an important topic, and our patients are increasingly paying attention to diet and exercise methods of optimizing their cancer treatments. At a recent think tank meeting hosted by David Crawford, our featured guest speaker was actually an accomplished lawyer who, in his late 80s, has taken up the cause of fighting sarcopenia with aging and put his research into a book for lay audiences called “Choosing the Strong Path.” You can find this book under the author's name, Fred Bartlit. It might be a good reference for yourself or a patient asking about this topic.
- *To the Future…* I am pleased to see another innovation paper from Professor Gundeti and his group from the University of Chicago, Adamic et al^8^. They bear the distinction of having submitted the very first paper to peer review in our new journal, and it appeared in our initial March 2020 issue. For this paper, the group presents their technique and a small cohort that underwent robotic‐assisted ureterocalycostomy in the pediatric population. This is a consideration for patients with heavily scarred lower pole and possibly as a revision technique. In our second innovation paper, Ohtaka et al. from Japan discuss a multi‐institutional experience of patients with malignant ureteral obstruction (extrinsic) who required stenting and compared a metallic product versus standard polyurethane products^9^. The patency was favorable for metallic stents, and the study shows some interesting analysis of “workload” scores for placing the different stents.
- *Videos, Editorials, and Research Communications…* As our journal grows, we have added content such as a video to accompany the ureterocalycostomy paper. Please see our YouTube account for *BJUI Compass* where we will post videos linked to articles as well as video lectures related to our content. For editorials, we have the Kukreja editorial mentioned on bladder cancer biomarkers. A second editorial from Uribe et al^10^ discusses a recent paper that proposes a more “surgical” definition of cure after brachytherapy, and they use this new standard with their database and explore this emerging concept that a PSA of ≤0.2 at 48 months post implant is a reasonable definition of cure. Finally, we also have a new short‐form submission format called “Research Communication.” These are 1,000 word articles with six or less references, see our author instruction page. In the communication by Beyer et al^11^, take us back to the ongoing COVID‐19 pandemic and explore the topic of delaying treatment for kidney cancer. The paper is fast in turnaround from concept to data with its use of Twitter to generate survey data, and its use of the CHERRIES method—checklist for reporting results of internet E‐surveys.

On a house‐keeping note, I want to welcome another member to our editorial board: Dr Michael B. Williams, an associate professor of urology at Eastern Virginia Medical School, and a partner with Urology of Virginia. Dr Williams did his residency at EVMS and fellowship at MD Anderson Cancer Center. He will be helping us with urologic oncology papers as well as advanced/metastatic prostate cancer topics.

**FIGURE 6:**
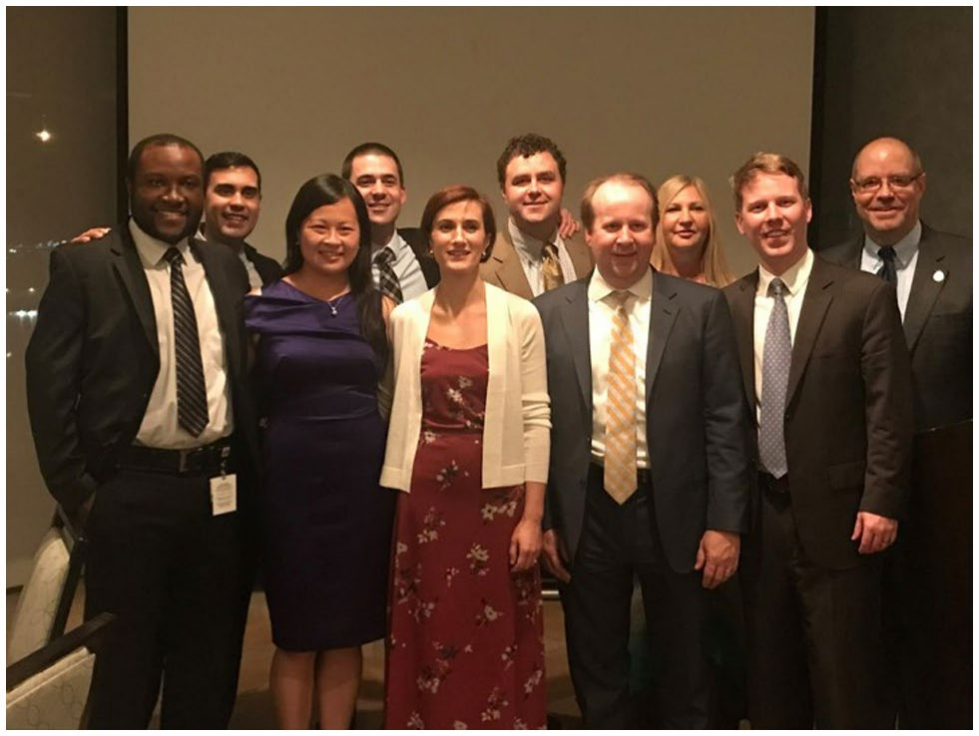
The 2017 version of the visiting professor with your editor handling the cases from the EVMS residents, and Kurt McCammon (far right) the Chair

**FIGURE 7:**
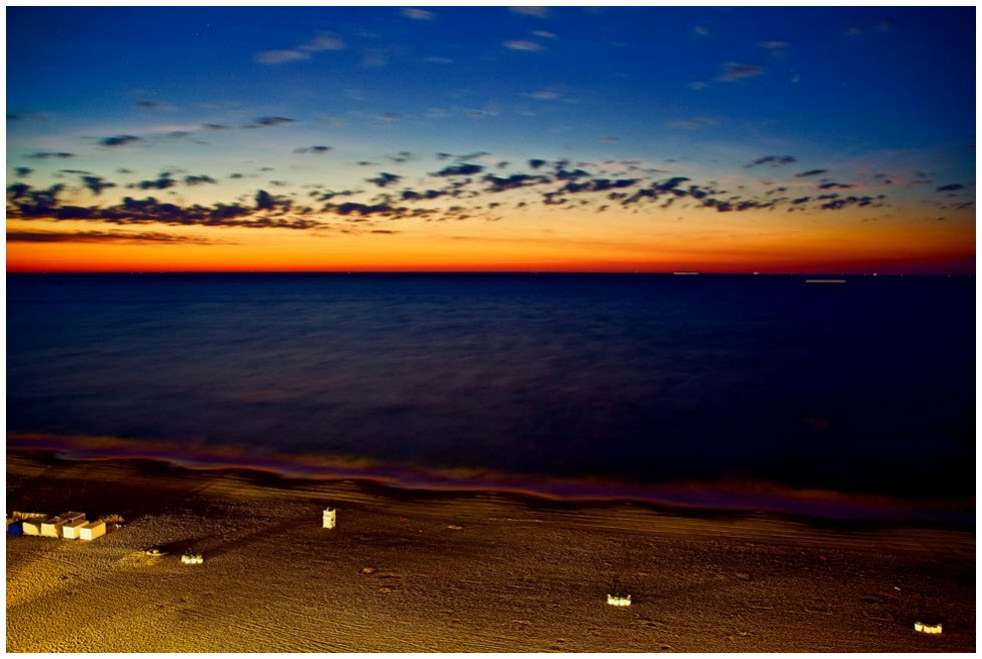
EVMS and Norfolk sit on the inward bay/harbour part of the region to the West, but a short drive away is Virginia Beach with fantastic sunrises over the Atlantic Ocean

Bringing it full circle will be Figures 6 and 7, my return to Norfolk as the Visiting Professor. I do not recall the residents throwing me any curve balls like we threw to Dr Menon in 2001. They wanted a graduation talk on a non‐medical topic, so it will be no surprise that I talked about my experiences combining photography with academic urology.
